# Age-Dependent Differences in the Rate and Symptoms of TIA Mimics in Patients Presenting With a Suspected TIA to a Neurological Emergency Room

**DOI:** 10.3389/fneur.2021.644223

**Published:** 2021-02-15

**Authors:** Franziska Maria Ippen, Fabian Walter, Christian Hametner, Christoph Gumbinger, Simon Nagel, Jan C. Purrucker, Sibu Mundiyanapurath

**Affiliations:** Department of Neurology, University Hospital Heidelberg, Heidelberg, Germany

**Keywords:** transient ischemic attack, stroke, TIA mimics, stroke mimics, age, elderly patients

## Abstract

**Background:** Transient ischemic attack (TIA) needs further diagnostic evaluation to prevent future ischemic stroke. However, prophylaxis can be harmful in elderly if the diagnosis is wrong. We aimed at characterizing differences in TIA mimics in younger and older patients to enhance diagnostic accuracy in elderly patients.

**Methods:** In a dedicated neurological emergency room (nER) of a tertiary care University hospital, patients with transient neurological symptoms suspicious of TIA (<24 h) were retrospectively analyzed regarding their final diagnoses and their symptoms. These parameters were compared between patients aged 18–70 and >70 years using descriptive, univariable, and multivariable statistics.

**Results:** From November 2018 until August 2019, 386 consecutive patients were included. 271 (70%) had cardiovascular risk factors and all patients received cerebral imaging, mostly CT [376 (97%)]. There was no difference in the rate of diagnosed TIA between the age groups [85 (46%) vs. 58 (39%); *p* = 0.213].TIA mimics in the elderly were more often internal medicine diseases [35 (19%) vs. 7 (5%); *p* < 0.001] and epileptic seizures [48 (26%) vs. 24 (16%); *p* = 0.032] but less often migraine [2 (1%) vs. 20 (13%); *p* < 0.001]. The most frequent symptoms in all patients were aphasia and dysarthria [107 (28%) and 92 (24%)]. Sensory impairments were less frequent in elderly patients [23 (11%) vs. 54 (30%); *p* < 0.001]. Impaired consciousness and orientation were independent predictors for TIA mimics (*p* < 0.001) whereas facial palsy (*p* < 0.001) motor weakness (*p* < 0.001), dysarthria (*p* = 0.022) and sensory impairment (*p* < 0.001) were independent predictors of TIA.

**Conclusion:** TIA mimics in elderly patients are more likely to be internal medicine diseases and epilepsy compared to younger patients. Excluding internal medicine diseases seems to be important in elderly patients. Facial palsy, motor weakness, dysarthria and sensory impairment are associated with TIA.

## Introduction

Up to 70% of transient neurological symptoms without acute cerebral infarction have underlying causes that mimic the signs but are distinct to transient ischemic attacks (TIA) and are therefore called TIA mimics (1–3). These mimics need to be properly distinguished from a potential TIA by the examining clinician, first and foremost for initiating the correct therapy for the patient, but also because a mistakenly initiation of secondary prevention therapies can yield potentially serious therapeutic consequences (4–7). Especially in elderly patients, a primary prevention with aspirin (due to misdiagnosis) has been shown to be associated with an increased risk for gastrointestinal bleeding and did not result in a considerable risk reduction of cardiovascular events or extension in overall survival (4–7).

This becomes even more important in western countries due to the increasing percentage of aged people in the population (8). So far, TIA mimics have been studied (2, 3, 9, 10) but not in an age-dependent manner. Some previous publications present infections as potential cause of TIA mimics, but they do not differentiate in which age these infections occur. We hypothesized that TIA mimics could be different in younger vs. older patients requiring a different approach towards differential diagnoses. If the diagnostic strategy does not include all differential diagnoses, some might be overlooked, especially if they only occur in certain age groups and are not commonly described as TIA mimics. In order to analyze an age-dependent distribution of TIA mimics in comparison to diagnosed TIAs, we conducted a cross-sectional study of consecutive patients who presented to our neurological emergency room with transient neurological symptoms suggestive of a TIA. We aimed at answering three questions: (1) Does the etiology differ in TIA mimics of older and younger patients? (2) Which symptoms are most frequent in elderly patients with transient neurological symptoms? (3) Which symptoms can predict TIA mimics in elderly patients?

## Methods

### Study Design, Setting, and Eligibility Criteria

This is a retrospective cross-sectional study of patients with transient neurological symptoms, who were admitted to the neurological emergency room (nER) at our tertiary care University Hospital. In our nER only patients with neurological symptoms are treated and are seen by a neurology resident with at least 2 years of training. Prehospital triage is excellent as ambulances have been familiar to this system for many years. Moreover, a senior neurologist is available for discussion at all times. In this study, data from our hospital database covering a period of 10 months, ranging from November 2018 to August 2019, were analyzed. Analyses were approved by the local ethics committee (S-637/2019) and the requirement for informed consent was waived.

We included consecutive patients ≥18 years with transient neurological symptoms that completely resolved within a time period of ≤24 h and were ascertainable by the National Institutes of Health stroke scale (NIHSS) (11). In case symptoms persisted beyond the presentation in the nER, patients were still included in this study as long as the symptoms did not persist longer than 24 h and adequate follow-up documentation was available (for example, discharge letters from other wards within Heidelberg University Hospital). All patients had to have a suspected TIA or at least a TIA must have been one of the suspected differential diagnoses. Therefore, patients with witnessed tonic-clonic seizures and post-ictal neurological symptoms were not included in the study cohort. As we aimed at investigating an age-related distribution of TIAs and TIA mimics, the severity of symptoms was not assessed. Instead, the presence of symptoms, which could be found on the NIHSS, was recorded on a dichotomized basis, whether they were present before the patient presented at the neurological emergency room and/or at the time of the initial neurological examination. A formal NIHSS score was not required, the presence of symptoms was extracted from the discharge letter. In case patients were diagnosed with a TIA, they did not have an acute infarction on CT or MRI, in line with the definition of the AHA/ASA (12). Some of the transient neurological symptoms were later confirmed to be ischemic stroke, these patients were included in the analysis as well. All patients received a detailed physical examination, diagnostic imaging (either CT, MRI or both) and blood analysis (electrolytes, kidney function, infection parameters, blood count, coagulation). After treatment in the nER, patients were either admitted to a ward at the tertiary care hospital, transferred to another hospital or discharged with recommendations for outpatient care. Depending on their destination, some patients received a more detailed diagnostic workup as Doppler sonography, echocardiography, ECG monitoring and EEG.

We predefined neurological diseases of special interest as TIA/ischemic stroke, migraine and epilepsy. Less frequent diseases were categorized as “other diagnoses.” Internal medicine diseases were analyzed as a group if the diagnosis was documented in >5 cases as we wanted to present the most frequent TIA mimics. Diseases that were less frequent were also categorized as “other diagnoses.”

Patients were stratified into different age groups: patients ranging in age from 18 to 70 years and patients that were older than 70 years in accordance to the age groups used in the ASPREE trial (4–7).

Final diagnosis was made by the treating physician in the nER in accordance to the local standard operating procedures or after consultation of a senior neurologist. All physicians had several years of neurological training. Sometimes, the final diagnosis was not especially stated in the discharge letter, especially if patients were discharged to outpatient care. These cases were independently reviewed and a *post hoc* analysis was performed by two authors (a neurology resident with 3 years of neurological experience and a neurology consultant with >10years of experience). In case of differing diagnoses, cases were discussed in detail and a consensus reading was achieved.

### Variables and Statistical Analyses

Standard descriptive statistics were used to analyze patients‘ demographic data, their previous vascular-related and neurological comorbidities, vascular risk factors, previous medication with antithrombotic medication, statins or anticonvulsant drugs as well as their diagnosis at time of discharge, such as neurological diagnosis, as well as internal medicine diagnoses or if the final diagnosis remained uncertain. Furthermore, standard descriptive statistics were used to investigate the distribution of patients among all analyzed age groups being discharged, admitted to a neurological or internal medicine ward or even external hospital or patients who were discharged against medical advice.

Fisher's exact tests and Pearson's chi-square tests were used to evaluate the association of possible TIA mimics diagnoses among different age groups, depending on the number of patients. In order to calculate the association of specific NIHSS-related symptoms with the occurrence of a TIA/ischemic stroke, binary logistic regression analyses were performed for multivariable analysis including known predictors of stroke. TIA/ischemic stroke was defined as the dependent variable, age (1 = 18–70; 2= >70); diabetes mellitus, hypertension, dyslipidemia, nicotine abuse, atrial fibrillation, coronary heart disease, peripheral arterial occlusive disease, history of ischemic stroke, history of transient ischemic attack, and one of each listed NIHSS symptom were defined as independent variables in this model. All statistical tests were two sided, and *p*-values of < 0.05 were considered to be significant. The analyses were carried out with SPSS 26.0 (IBM SPSS Statistics, Armonk, NY).

## Results

From a cohort of 7,396 patients who presented to the neurological ER of Heidelberg University Hospital between November 2018 to August 2019, we extracted a total of 386 consecutive patients who presented with transient neurological symptoms ascertainable with the NIHSS that resolved within less or equal to 24 h. Baseline characteristics of the study population are presented in Table 1 and Supplementary Table 1.

**Table 1:** Baseline characteristics of patients (*n*, %).

	**Total (*n* = 386)**	**18–70 years (*n* = 178)**	**>70years (*n* = 208)**	***p*-value**
Female	199 (52 %)	78 (44%)	121 (58%)	0.005
**Cardiovascular risk factors**				
- Diabetes mellitus	83 (22%)	30 (17%)	53 (26%)	0.040
- Hypertension	217(56%)	72 (40%)	145 (70%)	<0.001
- Dyslipidemia	64 (17%)	17 (10%)	47 (23%)	0.001
- Current smoker	51 (13%)	34 (19%)	17 (8%)	0.002
- Atrial fibrillation	77 (20%)	11 (6%)	66 (32%)	<0.001
**Pre-existing vascular comorbidities**				
- Coronary heart disease	82 (21%)	15 (8%)	67 (32%)	<0.001
- Peripheral artery disease	14 (4%)	1(1%)	13 (6%)	0.003
- Ischemic stroke	68 (18%)	17 (10%)	51 (25%)	<0.001
- TIA	23 (6%)	4 (2%)	19 (9%)	0.004
**Pre-existing other neurologic comorbidities**				
- Dementia	29 (8%)	2 (1%)	27 (13%)	<0.001
- History of seizure	16 (4%)	5 (3%)	11 (5%)	0.307
- History of brain tumor	8 (2%)	2 (1%)	6 (3%)	0.296
**Diagnostic imaging – no. (%)**				
- CT	376 (97%)	169 (95%)	207 (99.5%)	0.005
- CTA	100 (26%)	40 (23%)	60 (29%)	0.154
- CTP	5 (1%)	3 (2%)	2 (1%)	0.665
- MRI	51 (13%)	43 (24%)	8 (4%)	<0.001
- CT + MRI	41 11%)	34 (19%)	7 (3%)	<0.001
Ultrasound of intra- & extracranial arteries	105 (27%)	60 (34%)	45 (22%)	0.008
EEG	33 (9%)	17 (10%)	16 (8%)	0.515

*CT, computed tomography; CTA, CT-angiography; CTP, CT-perfusion; EEG, electroencephalogramm; MRI, magnetic resonance imaging; TIA, transient ischemic attack*.

An almost equal percentage of female and male patients presented at our nER. Comparing patients in a younger age group (18–70) and elderly patients (>70 years), all vascular risk factors and pre-existing vascular comorbidities (diabetes mellitus, hypertension, dyslipidemia, atrial fibrillation; coronary heart disease, peripheral arterial occlusive disease, history of ischemic stroke or transient ischemic attack) were significantly different between the two age groups. Elderly patients more often took single antiplatelet therapy [80 (39%) vs. 38 (21%); *p* < 0.001], therapeutic anticoagulation [67 (32%) vs. 16 (9%); *p* < 0.001] and statins [82 (39%) vs. 25 (14%); *p* < 0.001], but no differences were observed in regards to dual antiplatelet therapy [6 (3%) vs. 1 (1%); *p* = 0.130] and antiepileptic medication [19 (9%) vs. 10 (6%); *p* = 0.246]. All patients examined received diagnostic imaging. While the rate of CT-scans was not statistically different between elderly and younger patients [207 (100%) vs. 169 (95%); *p* = 0.154], cranial MRIs were less frequently conducted in elderly patients compared to the younger patient population ranging from 18 to 70 years in age [8 (4%) vs. 43 (24%); *p* < 0.001].

**Figure 1:**
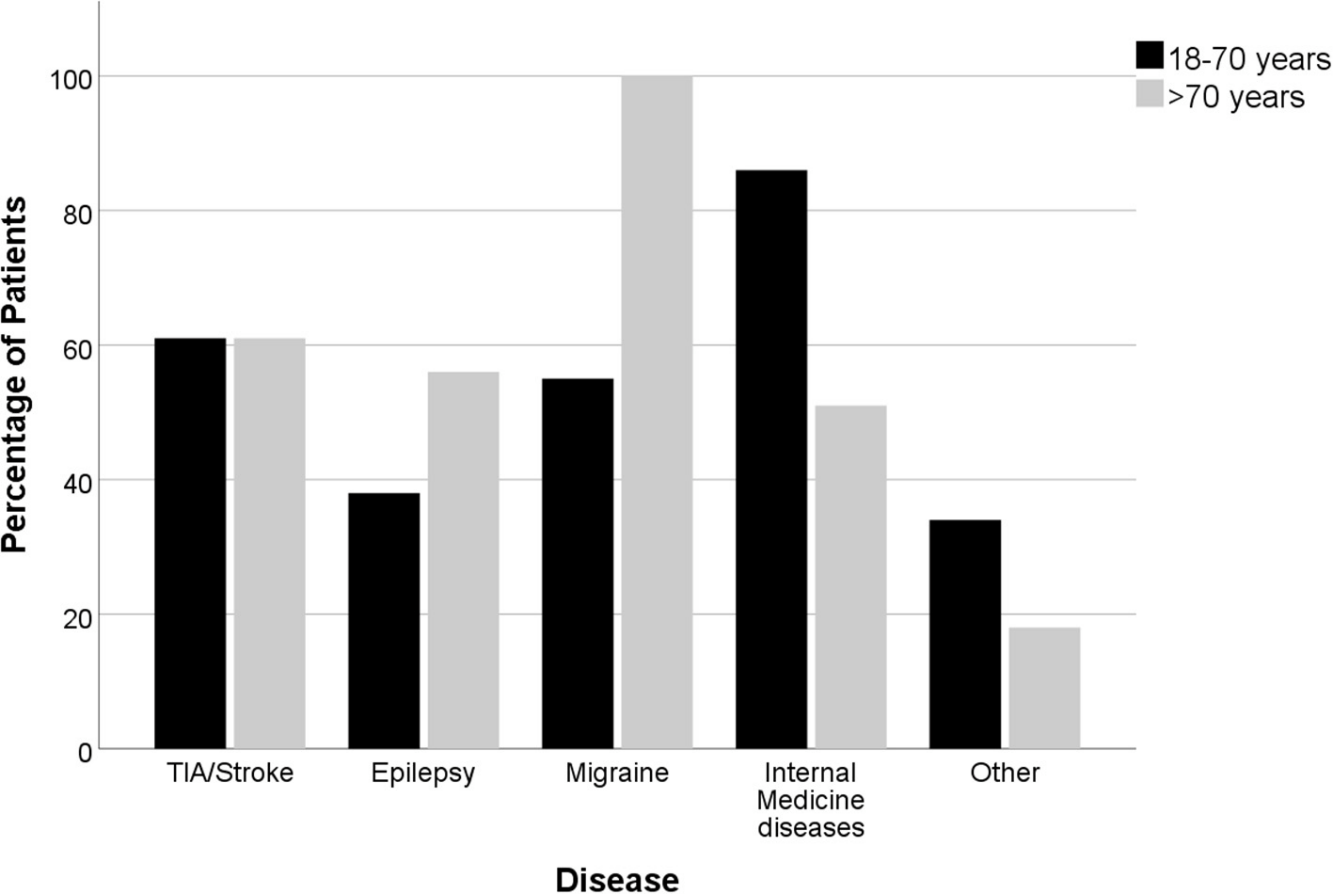
Distribution (percentage) of the diagnoses according to age (*n*= 178 age 18–70 years; *n* = 208 age >70 years).

A final diagnosis at the time of discharge and/or transferal to other hospitals was made in 335 (87%) cases, while in 51 (13%) patients, the final diagnosis remained unclear (Table 2, Figure 1). In case a final diagnosis was not especially stated in the discharge letter, the cases were independently reviewed and a *post hoc* analysis was performed by two authors (a neurology resident with 3 years of neurological experience and a neurology consultant with >10 years of experience). In total, this *post hoc* analysis was performed for 80 cases (21% of the study population).

**Table 2:** Diagnosis at discharge.

**Diagnosis**	**Total (*n* = 386)**	**18–70 years (*n* = 178)**	**>70 years (*n* = 208)**	***P*-value**
TIA	143 (43%)	58 (39%)	85 (46%)	0.213
Ischemic stroke	15 (5%)	11 (7%)	4 (2%)	0.031
Epileptic seizure	72 (22%)	24 (16%)	48 (26%)	0.032
Migraine attack	22 (7%)	20 (13%)	2 (1%)	<0.001
**Internal medicine diseases**				
- Infection	9 (3%)	2 (1%)	7 (4%)	0.308
- Dehydration	23 (7%)	2 (1%)	21 (11%)	<0.001
- Syncope	10 (3%)	3 (2%)	7 (4%)	0.521
Other diagnoses	41 (12%)	29 (20%)	12 (7%)	<0.001
Uncertain diagnosis	51 (13%)	29 (16%)	22 (11%)	0.098

*TIA, transient ischemic attack*.

Considering all patients with a final diagnosis, 42.7% of patients were diagnosed with a transient ischemic attack, 4.5% with an ischemic stroke and 52.8% with a TIA mimic.

In elderly patients, the following diagnoses were more common: Epileptic seizures [48 (26%) vs. 24 (16%); *p* = 0.032] and internal medicine diagnosis [35 (19%) vs. 7 (5%); *p* < 0.001], especially dehydration [21 (11%) vs. 2 (1%); *p* < 0.001]. Diagnoses that were less frequent included migraine attacks [2 (1%) vs. 20 (13%); *p* < 0.001], other diagnosis [12 (7%) vs. 29 (20%); *p* < 0.001] and ischemic strokes [4 (2%) vs. 11 (7%); *p* = 0.031]. The included migraine patients were admitted with suspected TIA. Consequently, all patients received a CT-scan. A CTA was added in 3 cases, ultrasound of the intra- & extracranial arteries was performed in 3 cases, an EEG was carried out in 1 patient diagnosed with a migraine. Of all 22 examined migraine patients in this cohort, five patients received an MRI. These patients were all in the younger patient population investigated and presented with a new kind of headache and one or a combination of the following symptoms: Dysarthria, Aphasia, impaired orientation, visual field deficiency and/or sensory deficits. Migraine was diagnosed if typical clinical symptoms developed or if cerebral ischemia was excluded with MRI in patients who were symptomatic for several hours. The most frequent diagnoses in the group of other diagnoses were functional neurological disorders and transient global amnesia (TGA), which were both less frequent in elderly patients [TGA: 2 (1%) vs. 7 (5%); *p* = 0.084; functional neurological symptoms: 0 (0%) vs. 7 (5%); *p* = 0.003, Supplementary Table 2]. TIA mimics were almost equally distributed among both age groups [97 (52%) vs. 80 (54%), respectively].

In regards to NIHSS-ascertainable symptoms, the majority of all patients presented to the nER with aphasia and dysarthria [107 (28%) and 92 (24%), respectively], followed by an altered level of consciousness {[84 (22%)], sensory deficits [77 (20%)] and an impaired orientation [69 (18%)], see Table 3}. Overall, 55.4% presented with one NIHSS-ascertainable symptom only. Only sensory impairment was significantly different between the age groups [23 (11%) in elderly patients vs. 54 (30%) in younger patients; *p* < 0.001].

**Table 3:** Distribution of symptoms according to the National Institute of Health Stroke Scale.

	**Total (*n* = 386)**	**18–70 years (*n* = 178)**	**>70 years (*n* = 208)**	***P*-value**
Impaired level of consciousness	84 (22%)	34 (19%)	50 (24%)	0.241
Impaired orientation	69 (18%)	33 (19%)	36 (17%)	0.753
Impaired completion of tasks	11 (3%)	4 (2%)	7 (3%)	0.510
Eye movement deviation	15 (4%)	6 (3%)	9 (4%)	0.628
Visual field deficiency	23 (6%)	14 (8%)	9 (4%)	0.143
Facial paresis	42 (11%)	16 (9%)	26 (13%)	0.269
Paresis extremities	127 (33%)	53 (30%)	74 (36%)	0.227
Limb ataxia	10 (3%)	5 (3%)	5 (2%)	0.803
Sensory impairment	77 (20%)	54 (30%)	23 (11%)	<0.001
Aphasia	107 (28%)	46 (26%)	61 (29%)	0.446
Dysarthria	92 (24%)	38 (21%)	54 (26%)	0.289
Extinction and inattention	3 (1%)	0 (0%)	3 (1%)	0.108

*Paresis of extremities were collapsed into one row*.

With a focus on the five most frequently observed symptoms, sensory impairment and dysarthria were particularly frequent in TIA patients (61% and 49%, respectively), whereas an impaired level of consciousness, an impaired orientation and aphasia were more common among TIA mimics (85%, 85%, and 53%, respectively)- especially among patients presenting with suspected epileptic seizures (50%, 41%, and 28%, respectively, Supplementary Table 3).

Speech disorders, i.e. aphasia or dysarthria, were present in all different diagnoses with differences between the age groups regarding the total number (Supplementary Figure 1). However, there was no statistically significant difference in the rate of patients compared between the age groups.

Binary logistic regression analysis adjusting for cardiovascular risk factors, cardiovascular comorbidities and age revealed that symptoms significantly correlating with the final diagnosis of TIA/ischemic stroke were the presence of a facial paresis [OR 2.37; 95% CI (1.13–4.94); *p* = 0.022], paresis of the extremities [OR 5.10; 95% CI (2.78–9.36); *p* < 0.001], sensory impairment [OR 3.32; 95% CI (1.76–6.28); *p* < 0.001] or the presence of dysarthria [OR 1.85; 95% CI (1.09–3.12); *p* = 0.022]. None of the individual cardiovascular risk factors were independent predictors of TIA, smoking showed a statistical trend [OR 1.89; 95% CI (0.96–3.72); *p* = 0.066].

Symptoms which were significant negative predictors of TIA/stroke were impaired level of consciousness [OR = 0.14; 95% CI (0.07–0.28); *p* < 0.001] and impaired orientation [OR = 0.16; 95% CI (0.07–0.35); *p* < 0.001]. In a subgroup analysis with only elderly patients, no differences to the analysis with all patients were found.

Overall, most patients [181 (47%)] were discharged within 24 h after their presentation to the nER, followed by patients who were admitted to one of our neurological wards for a more detailed diagnostic workup [124 (32%)] and by patients who were transferred to neurological wards of external hospitals [44 (11%)]. Twenty-nine (8%) patients were discharged against medical advice and 8 (2%) were transferred to an internal medicine ward. A marked difference toward elderly patients being transferred to external neurological wards was noticeable [36 (17%) vs. 8 (5%); *p* < 0.001; Supplementary Table 4].

## Discussion

In our study, we analyzed an age-dependent distribution of TIA mimics in comparison to diagnosed TIAs in a cross-sectional study of patients who presented in our nER with transient neurological symptoms ascertainable with the NIHSS. We demonstrated that elderly patients (>70 years) have different TIA mimics compared to younger patients (18–70 years). Elderly patients had significantly more often internal medicine diseases and epileptic seizures and significantly less frequently migraines. Besides sensory impairment, there was no significant difference between the age groups regarding the frequency of symptoms. While facial paresis, paresis of the extremities, sensory impairment and dysarthria were significant positive predictors for TIA, impaired level of consciousness and impaired orientation were negative predictors. There was no difference in age subgroups if adjusted for cardiovascular risk factors.

Our study presents a representative cohort in which the percentage of TIA mimics was 53% and almost equally distributed in patients aged 18–70 compared to patients older than 70 years. These percentage of patients diagnosed with TIA mimics are in line with various previous publications, which have shown that up to 70% of patients with temporary neurological symptoms have TIA mimics (2, 3, 13–15). Moreover, known symptoms predicting a TIA such as motor weakness and sensory deficits were independent predictors of a TIA in our study as well as previously published negative independent predictors as disorientation and impaired consciousness (16).

Regarding TIA mimics, many of the etiologies found in our study (e.g., epilepsy, migraine) have been reported in other studies as well (2, 16). However, there is little data on neurological symptoms on internal medicine diseases, especially dehydration, even though it is a frequent complaint of elderly patients (17). In our study, we found that dehydration can also cause neurological symptoms like aphasia and dysarthria which are recognized as symptoms suggestive for a definitive TIA (16). It seems to make sense to exclude internal medicine diagnoses before making the final diagnosis of an TIA, especially in elderly people. This is supported by a small study examining 21 patients with isolated aphasia (18). The authors found that toxic/metabolic disturbances were a common cause (eight patients, 39%). While we agree with the statement that aphasia can be caused by internal medicine diseases, we disagree with their conclusion that the likelihood for ischemia is low. Our study showed that patients with aphasia and dysarthria most likely suffer from TIA/stroke. If the final diagnosis in these cases remains unclear, an ischemic etiology is the most likely and should lead to further diagnostic evaluation and treatment. In a patient with ongoing severe aphasia, additional diagnostics, e.g., CT-perfusion or MRI, might help to distinguish stroke/TIA from mimics although a CT-perfusion cannot exclude cerebral ischemia.

A recent systematic review has found that TIA mimics (especially multiple sclerosis and functional neurological symptoms) are more frequent in younger patients (1). While this could not be entirely reproduced in our study regarding all TIA mimics, it was true for some diagnoses. Especially functional neurological symptoms and TGA were more frequent in younger patients (keeping in mind, that younger patients were defined as ≤70 years. In addition, the dichotomization dilutes this effect that might have been found if more age groups had been used.

In the near future, computational models might be a promising approach in order to distinguish between TIA and mimics: a recent predictive analytics model has taken clinical and imaging elements considered for the diagnosis of TIA, a TIA mimic or a minor stroke into account and developed two different multinomial models incorporating feature selection for differentiating between these potential diagnoses in a cohort with a similar size. Although this approach seems promising, further improvements like noise filters are still warranted in order to improve the quality of these predictive models (9).

In summary, the differentiation of a TIA/stroke vs. a potential TIA mimic warrants the evaluation of an experienced clinician taking several aspects into account. Age, cardiovascular risk factors and comorbidities as well as the onset and progression of symptoms can be an important factor for the initial distinction, but a high quality physical examination, cerebral imaging such as a CT-scan or an MRI, laboratory results (including infection parameters, kidney function, electrolytes and blood count), are crucial to assess. Especially in elderly patients, more TIA mimics than previously reported are internal medicine diseases and have to be considered as potential etiology for transient neurologic symptoms. In some cases, consultation with a specialist for internal medicine might be necessary.

## Limitations of this Study

This study has inherent limitations based on its retrospective nature and all conclusions have to be interpreted with caution and considered to be exploratory. All results have to be replicated in a prospective trial before they could potentially be translated into clinical practice. Furthermore, this is a monocentric study focusing on patients admitted to a nER, and nERs are not a standard subdivision of ERs in all hospitals or countries. On the other hand, this setting allowed us to have a highly specialized team, increasing diagnostic accuracy. The study also has limitations in regards to the symptoms assessed as only temporary symptoms ascertainable with the NIHSS were included in this study. Other symptoms such as vertigo, paresthesias etc. were not included, which might alter the implemented analyses. Furthermore, the dichotomization in two age groups cannot illustrate a more detailed view on patients across all age groups and is therefore prone to oversimplification. We also restricted our cohort to patients with the initial suspicion of a TIA, which has to be kept in mind when comparing our results to studies that included all patients with transient neurological symptoms. In addition, reviewing the cases with unspecified diagnosis might have caused a distortion to the results which we reduced by performing the revision by two authors independently. We also cannot exclude cases of misdiagnosis, as the majority of diagnoses were made based on patient history, clinical examination and CT-scan.

## Data Availability Statement

The raw data supporting the conclusions of this article will be made available by the authors, without undue reservation.

## Author Contributions

FI, FW, and SM concepted and designed the study and obtained ethical approval, carried out the data acquisition, performed the statistical analysis, verified the statistical analysis, and interpreted the obtained data and wrote, reviewed and revised the manuscript. FI and SM developed the methodology of the study. FW and SM designed the figures and tables. CH, CG, SN, and JP reviewed and revised the manuscript and aided in interpreting the results. The study was supervised by FI and SM. All authors contributed to the article and approved the submitted version.

## Conflict of Interest

The authors declare that the research was conducted in the absence of any commercial or financial relationships that could be construed as a potential conflict of interest.

## Supplementary Material

Supplementary Figure 1 Number of patients with speech disorders in the different diagnoses.

Supplementary Table 1 Further baseline characteristics.

Supplementary Table 2 Diagnoses in the group “other diagnoses”.

Supplementary Table 3 Distribution of diseases for the five most frequent symptoms.

Supplementary Table 4 Destination after discharge from the neurological ER.
