# Supplementary material for: Age-Dependent Differences in the Rate and Symptoms of TIA Mimics in Patients Presenting With a Suspected TIA to a Neurological Emergency Room
Source: Front Neurol. 2021 Feb 15;12:644223. doi: 10.3389/fneur.2021.644223 (PMC7917180; doi:10.3389/fneur.2021.644223)
Supplement: Supplementary Table 1 — Further baseline characteristics. [file Table_1.docx]

**Supplementary Table 1: Further baseline characteristics**

| **Previous medication – no. (%)** | **Total** | | **18-70 years** | | **>70 years** | | **p-value** |
| --- | --- | --- | --- | --- | --- | --- | --- |
| **Single antiplatelet therapy** | 118 | (30.6) | 38 | (21.3) | 80 | (38.5) | <0.001 |
| **ASS** | 108 | (28.0) | 35 | (19.7) | 73 | (35.1) | 0.001 |
| **Clopidogrel** | 9 | (2.3) | 3 | (1.7) | 6 | (2.9) | 0.436 |
| **Ticagrelor** | 1 | (0.3) | 0 | (0.0) | 1 | (0.5) | 0.354 |
| **Dual antiplatelet therapy** | 7 | (1.8) | 1 | (0.6) | 6 | (2.9) | 0.088 |
| **ASS + Clopidogrel** | 5 | (1.3) | 1 | (0.6) | 4 | (1.9) | 0.238 |
| **ASS + Ticagrelor** | 2 | (0.5) | 0 | (0.0) | 2 | (1.0) | 0.190 |
| **Therapeutic Anticoagulation** | 83 | (21.5) | 16 | (9.0) | 67 | (32.2) | <0.001 |
| **Dabigatran** | 6 | (1.6) | 3 | (1.7) | 3 | (1.4) | 0.847 |
| **Apixaban** | 26 | (6.7) | 3 | (1.7) | 23 | (11.1) | <0.001 |
| **Rivaroxaban** | 22 | (5.7) | 1 | (0.6) | 21 | (10.1) | <0.001 |
| **Enoxaparin** | 10 | (2.6) | 3 | (1.7) | 7 | (3.4) | 0.300 |
| **Phenprocoumon** | 12 | (3.1) | 4 | (2.2) | 8 | (3.8) | 0.367 |
| **Edoxaban** | 5 | (1.3) | 1 | (0.6) | 4 | (1.9) | 0.238 |
| **Heparin** | 1 | (0.3) | 0 | (0.0) | 1 | (0.5) | 0.354 |
| **Statin** | 107 | (27.7) | 25 | (14.0) | 82 | (39.4) | <0.001 |
| **Anticonvulsant** | 29 | (7.5) | 10 | (5.6) | 19 | (9.1) | 0.191 |
| **Lamotrigin** | 3 | (0.8) | 1 | (0.6) | 2 | (1.0) | 0.656 |
| **Levetiracetam** | 9 | (2.3) | 1 | (0.6) | 8 | (3.8) | 0.033 |
| **Carbamazepin** | 1 | (0.3) | 0 | (0.0) | 1 | (0.5) | 0.354 |
| **Gabapentin** | 6 | (1.6) | 1 | (0.6) | 5 | (2.4) | 0.145 |
| **Valproat** | 2 | (0.5) | 2 | (1.1) | 0 | (0.0) | 0.125 |
| **Lacosamid** | 1 | (0.3) | 0 | (0.0) | 1 | (0.5) | 0.354 |
| **Pregabalin** | 12 | (3.1) | 6 | (3.4) | 6 | (2.9) | 0.784 |
| **Phenytoin** | 1 | (0.3) | 1 | (0.6) | 0 | (0.0) | 0.279 |

**Supplementary Table 2: Diagnoses in the group “other diagnoses”**

| **Diagnosis** | **18-70 years**  **n/%** | | **> 70 years**  **n/%** | | **P-value** |
| --- | --- | --- | --- | --- | --- |
| **Hypoglycemia** | 1 | (0.7) | 0 | (0.0) | 0.445 |
| **Subarachnoid hemorrhage** | 1 | (0.7) | 0 | (0.0) | 0.445 |
| **Nerve damage** | 3 | (2.0) | 0 | (0.0) | 0.087 |
| **Transient global amnesia** | 7 | (4.7) | 2 | (1.1) | 0.084 |
| **Hypertensive crisis** | 3 | (2.0) | 2 | (1.1) | 0.659 |
| **Functional neurological symptoms** | 7 | (4.7) | 0 | (0.0) | 0.003 |
| **Intoxication** | 2 | (1.3) | 5 | (2.7) | 0.468 |
| **Unspecific Headache** | 2 | (1.3) | 0 | (0.0) | 0.197 |
| **Delirium** | 0 | (0.0) | 2 | (1.1) | 0.505 |
| **Other etiologies (hyperthermia, idiopathic intracranial hypertension, fatigue, COPD** | 3 | (2.0) | 1 | (0.5) | 0.327 |

| **Diagnosis** | **Impaired level of consciousness** | | | | | | **Impaired orientation** | | | | | | **Sensory impairment** | | | | | | **Aphasia** | | | | | | **Dysarthria** | | | | | |
| --- | --- | --- | --- | --- | --- | --- | --- | --- | --- | --- | --- | --- | --- | --- | --- | --- | --- | --- | --- | --- | --- | --- | --- | --- | --- | --- | --- | --- | --- | --- |
|  | Total  n / % | | 18-70 years  n / % | | > 70 years  n / % | | Total  n / % | | 18-70 years  n / % | | > 70 years  n / % | | Total  n / % | | 18-70 years  n / % | | > 70 years  n / % | | Total  n / % | | 18-70 years  n / % | | > 70 years  n / % | | Total  n / % | | 18-70 years  n / % | | > 70 years  n / % | |
| **TIA** | 8 | 11 | 4 | 15 | 4 | 9 | 7 | 13 | 4 | 15 | 3 | 11 | 37 | 61 | 20 | 48 | 17 | 89 | 43 | 43 | 17 | 40 | 26 | 46 | 43 | 52 | 18 | 51 | 25 | 52 |
| **Ischemic stroke** | 3 | 4 | 1 | 4 | 2 | 4 | 1 | 2 | 0 | 0 | 1 | 4 | 5 | 8 | 5 | 12 | 0 | 0 | 4 | 4 | 2 | 5 | 2 | 4 | 6 | 7 | 5 | 14 | 1 | 2 |
| **TIA mimics** | 63 | 85 | 22 | 81 | 41 | 87 | 46 | 85 | 22 | 85 | 24 | 86 | 19 | 31 | 17 | 40 | 2 | 11 | 52 | 53 | 24 | 56 | 28 | 50 | 34 | 41 | 12 | 34 | 22 | 46 |
| **Epileptic seizure** | 37 | 50 | 16 | 59 | 21 | 45 | 22 | 41 | 11 | 42 | 11 | 39 | 5 | 8 | 3 | 7 | 2 | 11 | 25 | 25 | 6 | 14 | 19 | 34 | 11 | 13 | 3 | 9 | 8 | 17 |
| **Migraine** | 0 | 0 | 0 | 0 | 0 | 0 | 1 | 2 | 1 | 4 | 0 | 0 | 7 | 11 | 7 | 17 | 0 | 0 | 9 | 9 | 9 | 21 | 0 | 0 | 4 | 5 | 2 | 6 | 2 | 4 |
| **Infection** | 2 | 3 | 0 | 0 | 2 | 4 | 0 | 0 | 0 | 0 | 0 | 0 | 0 | 0 | 0 | 0 | 0 | 0 | 3 | 3 | 1 | 2 | 2 | 4 | 3 | 4 | 1 | 3 | 2 | 4 |
| **Dehydration** | 8 | 11 | 0 | 0 | 8 | 17 | 9 | 17 | 0 | 0 | 9 | 32 | 0 | 0 | 0 | 0 | 0 | 0 | 6 | 6 | 1 | 2 | 5 | 9 | 9 | 11 | 2 | 6 | 7 | 15 |
| **Syncope** | 9 | 12 | 3 | 11 | 6 | 13 | 0 | 0 | 0 | 0 | 0 | 0 | 0 | 0 | 0 | 0 | 0 | 0 | 2 | 2 | 1 | 2 | 1 | 2 | 0 | 0 | 0 | 0 | 0 | 0 |
| **Other diagnosis** | 7 | 10 | 3 | 11 | 4 | 9 | 14 | 26 | 10 | 38 | 4 | 14 | 7 | 11 | 7 | 17 | 0 | 0 | 7 | 7 | 6 | 14 | 1 | 2 | 7 | 8 | 4 | 11 | 3 | 6 |
| **Uncertain diagnosis** | 10 | 12 | 7 | 21 | 3 | 6 | 15 | 22 | 7 | 21 | 8 | 22 | 16 | 21 | 12 | 22 | 4 | 17 | 8 | 7 | 3 | 7 | 5 | 8 | 9 | 10 | 3 | 8 | 6 | 11 |

**Supplementary Table 3: Distribution of diseases for the five most frequent symptoms**

**Supplementary Table 4: Destination after discharge from the neurological ER**

| **Patient management - no. (%)** | **All patients (n=386)** | | **18-70 years (n=178)** | | **> 70 years (n=208)** | | **P-value** |
| --- | --- | --- | --- | --- | --- | --- | --- |
| Discharge after < 24h | 181 | (46.9) | 90 | (50.6) | 91 | (43.8) | 0.181 |
| Admission to neurology unit at Heidelberg University Hospital | 124 | (32.1) | 66 | (37.1) | 58 | (27.9) | 0.054 |
| Admission to neurology unit at an external hospital | 44 | (11.4) | 8 | (4.5) | 36 | (17.3) | < 0.001 |
| Admission to internal medicine unit | 8 | (2.1) | 6 | (3.4) | 2 | (1.0) | 0.151 |
| Discharge against medical advice | 29 | (7.5) | 8 | (4.5) | 21 | (10.1) | 0.037 |
